# Diabetes-Related Complications: Mechanisms and Emerging Therapies

**DOI:** 10.3390/cells15141296

**Published:** 2026-07-20

**Authors:** Vijaya Prathigudupu, Minhyuk Lee, Isaac Uriarte, Gadiel So, Rabina Kaur, Sabrina Kaur, Fatima Ghacham, Vishwanath Venketaraman

**Affiliations:** College of Osteopathic Medicine of the Pacific, Western University of Health Sciences, Pomona, CA 91766, USA; vijaya.prathigudupu@westernu.edu (V.P.); minhyuk.lee@westernu.edu (M.L.); isaac.uriarte@westernu.edu (I.U.); gadiel.so@westernu.edu (G.S.); rabina.kaur@westernu.edu (R.K.); sabrina.kaur@westernu.edu (S.K.); fatima.ghacham@westernu.edu (F.G.)

**Keywords:** diabetes mellitus, reactive oxygen species, AGE-RAGE, inflammation, diabetic retinopathy, foot ulcers, nephropathy

## Abstract

Diabetes Mellitus (DM) is a metabolic disorder characterized by chronic hyperglycemia resulting from the body’s inability to produce insulin or effectively respond to it. In this review, we summarize the cellular and molecular mechanisms involved in common clinical manifestations of DM, including neuropathy, retinopathy, nephropathy, sensorineural hearing loss, cardiovascular disease, stroke, immune dysfunction, and chronic wounds. Common pathways explored in these dysfunctions include the polyol pathway, formation of advanced glycation end products (AGEs) and their interaction with the receptor for advanced glycation end products (RAGE), mitochondrial dysfunctions leading to overproduction of reactive oxygen species (ROS), and the chronic state of inflammation and inflammatory cytokines. Furthermore, we explore current and developing treatments for DM, along with more targeted therapies for the clinical manifestations of DM. In summary, this review gives a comprehensive overview of the pathways involved in the clinical manifestations of DM, and how these pathways are targets for therapeutics and treatments.

## 1. Introduction

Diabetes Mellitus (DM) is a chronic metabolic disorder characterized by impaired insulin production, secretion, or sensitivity, leading to hyperglycemia. An estimated 800 million individuals are affected worldwide, highlighting its immense public health burden [1]. Both genetic predisposition and environmental factors, including diet, physical inactivity, and obesity, contribute to the disruption of glucose homeostasis.

Glucose homeostasis is tightly controlled by the actions of β-cells in the pancreas. Following a meal, elevated blood glucose is detected, which triggers the secretion of insulin by β-cells in the pancreatic islets of Langerhans. Insulin promotes glucose uptake in peripheral cells, especially in skeletal and adipose cells, through the Glucose Transporter Type 4 (GLUT4) receptor, returning the body to normal blood glucose levels. Impairment in this endocrine pathway can lead to DM.

DM can be broadly classified into type 1 DM (T1DM), resulting in the autoimmune destruction of β-cells, or type 2 DM (T2DM), characterized by decreased insulin sensitivity or decreased insulin secretion, leading to sustained hyperglycemia [2].

Chronic hyperglycemia leads to non-enzymatic glycation of proteins, lipids, and nucleic acids, with one of the most important examples being glycation of hemoglobin (Hb) leading to the formation of HbA1c. This glycated hemoglobin can be detected in the blood for up to 3 months and is an important indicator for diagnosing and tracking the progression of diabetes [3]. Moreover, the glycation of molecules leads to the formation of advanced glycation end products (AGEs) that, when accumulated, can lead to oxidative stress, inflammation, and cellular and tissue damage [4].

**Figure 1:**
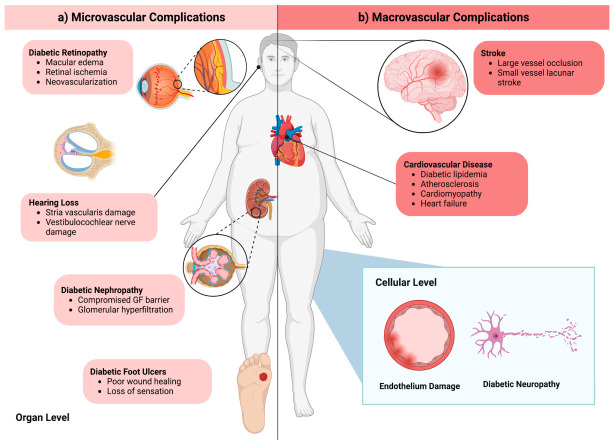
Overview of Diabetic-Induced Organ Damage. At the organ level, organs are damaged due to micro- and macrovascular complications. (**a**) Microvascular complications lead to notable damage such as diabetic retinopathy from macular edema, retinal ischemia, and neovascularization. Diabetic nephropathy is caused by a compromised glomerular filtration barrier and glomerular hyperfiltration. Diabetic foot ulcers arise from poor wound healing due to attenuated macrophage differentiation, and diabetic neuropathy causes loss of sensation. (**b**) Macrovascular complications include ischemic stroke from endothelial damage to either large vessels or small vessels leading to large vessel occlusions and lacunar strokes, respectively. Endothelial damage arising from a similar mechanism can cause a wide array of cardiovascular diseases, including atherosclerosis. Underlying these organ system damages are vascular endothelial dysfunction and peripheral neuropathy at the cellular level. Created in BioRender. Lee, M. https://BioRender.com/68zel5p (accessed on 21 May 2026).

Prolonged hyperglycemia also radically changes the nerves and vasculature in an organ, which ultimately impairs the organ’s functions. Such deleterious effects span multiple organ systems, contributing to microvascular complications such as retinopathy, nephropathy, and neuropathy, as well as macrovascular complications, including cardiovascular disease and stroke (Figure 1). It also impairs wound healing and the immune response, further increasing morbidity in affected individuals [5].

## 2. Materials and Methods

This study was conducted as a narrative review of the literature examining the complications of Diabetes Mellitus across multiple organ systems. PubMed and Google Scholar were searched for peer-reviewed articles on diabetes-induced organ complications and their underlying mechanisms, primarily published between 2015 and 2025. The search was expanded to include selected landmark mechanistic studies and key clinical investigations published between 1989 and 2026 when they provided important mechanistic or clinical context for the topics discussed.

Search terms included “diabetes mellitus,” “stroke+diabetes,” “diabetic retinopathy,” “diabetic neuropathy,” “diabetes+cardiovascular disease,” “diabetes+AGE+RAGE,” “diabetes+immune system,” “diabetes+wounds,” “diabetic nephropathy,” “diabetes+hearing loss,” and “diabetes management.” 

Articles were included based on their relevance to the scope of the review, with priority given to studies providing mechanistic insight, clinical relevance, or advances in therapeutic approaches. Articles that were not directly relevant to the scope of the review or did not contribute meaningful mechanistic, clinical, or therapeutic insight were excluded. The literature was synthesized and organized according to organ systems and common pathogenic mechanisms.

No generative artificial intelligence (GenAI) was used in the writing of this manuscript.

## 3. Cellular Mechanisms of Diabetes-Induced Damage

### 3.1. Vascular Damage and Endothelial Dysfunction

Vascular damage, such as atherosclerosis (AS) or peripheral arterial disease (PAD), is a common complication of DM that can burden other organ systems by compromising blood flow. A healthy endothelium of the blood vessels is critical for maintaining vascular homeostasis. However, in patients with DM, many hyperglycemia-induced cellular mechanisms, including oxidative stress, inflammation, and endothelial-to-mesenchymal transition, lead to endothelial dysfunction, which ultimately results in vascular damage. 

In hyperglycemia, an increase in metabolic flux through glycolysis and the tricarboxylic acid (TCA) cycle leads to the increased production of electron-donating byproducts, NADH and FADH_2_, which overloads the mitochondrial electron transport chain (ETC) and therefore increases inner mitochondrial membrane potential [6]. This ultimately increases the likelihood of ROS formation by excess reducing equivalents leaking and prematurely reducing the oxygen to superoxide in the mitochondria. Hyperglycemia has also been shown to stimulate protein kinase C (PKC) by increasing the production of diacylglycerol (DAG) [7]. PKC, in turn, phosphorylates NADPH oxidase (NOX) subunits, leading to higher production of ROS in diabetes patients. Inflammation is also one of the major processes involved in endothelial dysfunction, and hyperglycemia has been shown to activate multiple inflammatory pathways, which lead to the production of damaging cytokines (IL-1β, TNF-α) and adhesion molecules (VCAM-1, ICAM-1) [8]. A major cellular mechanism by which hyperglycemia promotes inflammation is via AGEs. A high glucose environment increases the production of AGEs, which bind to their receptor, RAGE, to activate multiple signal transduction pathways, ultimately activating NF-κB and the release of cytokines and adhesion molecules. Notably, NF-κB, known as the pro-inflammatory master switch, is activated by hyperglycemia [9]. NF-κB, in turn, promotes the transcription of the pro-inflammatory cytokine IL-1β, leading to inflammation and endothelial dysfunction. These inflammatory processes, in turn, derail vascular endothelial cells from maintaining their vascular homeostasis, resulting in endothelial dysfunction and vascular damage. This acts in a positive feedback loop, as NF-κB further increases the expression of RAGE, leading to a vicious cycle of chronic inflammation in patients with DM [10].

**Figure 2:**
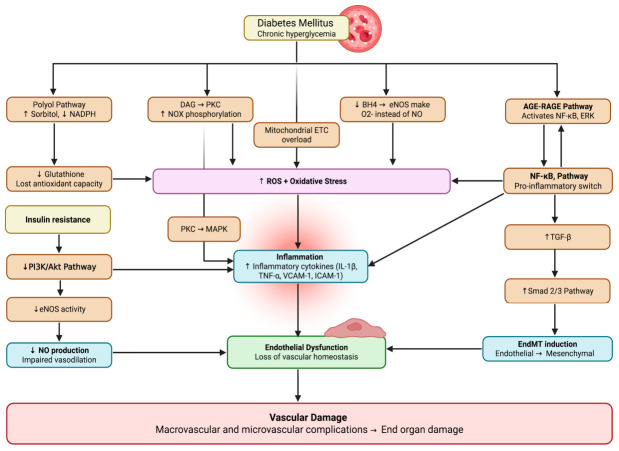
Cellular Mechanism of Endothelial Dysfunction in Diabetes Mellitus. Excess glucose funnels to the polyol pathway, increasing sorbitol; a subsequent decrease in NADPH leads to decreased glutathione, leading to oxidative stress. Excess glucose activates Protein Kinase C (PKC), leading to NADPH oxidase and Mitogen-Activated Protein Kinase (MAPK) activation, leading to an increase in ROS and Inflammation, respectively. Excess glucose leads to the production of AGEs, binding to their receptors to turn on nuclear factor kappa B (NF-κB), the pro-inflammatory master switch. NF-κB induces endothelial cells to a more fibrotic and mesenchymal phenotype by increasing TGF-β and inducing Smad 2/3 pathway. Insulin resistance decreases endothelial NO synthase (eNOS), impairing vasodilation and contributing to endothelial dysfunction. Inflammation, impaired vasodilation, and endothelial-to-mesenchymal transition (EndMT) collectively lead to endothelial dysfunction, damaging the vasculature and ultimately causing different organ-level complications seen in DM. Created in BioRender. Lee, M. https://BioRender.com/68zel5p (accessed on 21 May 2026).

Lastly, high glucose concentration eventually saturates hexokinase, causing glucose to be converted to sorbitol, a type of polyol, by aldose reductase using NADPH as a cofactor [6]. This depletes NADPH, preventing it from serving as a cofactor to regenerate glutathione (GSH), a crucial antioxidant, leading to decreased GSH production and a loss of antioxidant capacity. Moreover, hyperglycemia also depletes another cofactor, tetrahydrobiopterin (BH4), used by endothelial nitric oxide synthase (eNOS) to produce nitric oxide (NO) for vasodilation and maintenance of vascular homeostasis [11]. Without BH4, eNOS in endothelial cells produces superoxide instead of NO, leading to the production of ROS. These pathways all lead to the overproduction of ROS, leading to oxidative stress that contributes to endothelial dysfunction and vascular damage (Figure 2).

Insulin resistance, another hallmark of T2DM, also plays a role in disrupting vascular homeostasis by inhibiting the production of NO via eNOS, which is activated by the PI3K/Akt pathway [12,13]. While insulin normally activates the PI3K/Akt pathway to activate eNOS to produce NO in endothelial cells, resistance to insulin suppresses this pathway and leads to inflammation. Downregulation of the PI3K/Akt pathway is also responsible for the release of pro-inflammatory mediators such as VCAM-1, further impairing vascular homeostasis. Furthermore, hyperglycemia-induced PKC activation due to increased de novo synthesis of DAG, discussed earlier in the context of ROS production, also activates the MAPK pathway. This leads to the release of VCAM-1, ICAM-1, E-selectin, and plasminogen activator inhibitor-1 (PAI-1), all leading to increased inflammation and endothelial dysfunction [13].

Lastly, vascular homeostasis is disrupted when endothelial cells in the vasculature, under hyperglycemia-induced stressors, transition to a mesenchymal phenotype, a process known as endothelial-to-mesenchymal transition (EndMT) [14]. An in vitro study using human umbilical vein endothelial cells (HUVEC) showed that hyperglycemia induced EndMT, suggesting its implications in diabetes complications [15]. The high glucose environment induced morphological features of mesenchymal cells and increased mRNA levels of TGF-β1. Increased TGF-β signaling in turn activates the canonical Smad 2/3 pathway. Smad 2/3 is a transcription factor that increases the transcription of gene products that change endothelial cells to a more fibrotic and mesenchymal phenotype. This EndMT process is amplified by other hyperglycemia-induced processes, such as oxidative stress and inflammation, as they both increase TGF-β production via NF-κB and other pathways [14]. Furthermore, there are non-TGF-β pathways, such as endothelin-1 and Notch signaling, that get activated by hyperglycemia and induce EndMT independently of TGF-β effects. 

Of the current standard pharmacological therapies for DM, many have been shown to be protective against vascular damage complications. A recent review article by Liu et al. investigated each drug category used for the treatment of DM and determined that glucagon-like peptide-1 (GLP-1) receptor agonists, Metformin, and sodium-glucose cotransporter 2 (SGLT-2) inhibitors are highly effective in attenuating endothelial dysfunction in DM [12]. These medications, via different mechanisms of action, reduce inflammation, reduce oxidative stress and/or increase nitric oxide production, thus maintaining endothelial homeostasis.

### 3.2. Diabetic Neuropathy

**Figure 3:**
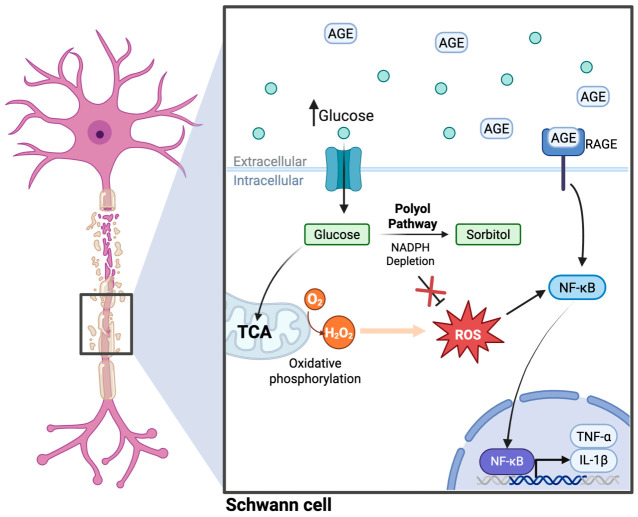
Myelin Degeneration in Diabetic Neuropathy. Pathways leading to myelin degeneration include activation of RAGE by binding of AGE, the intracellular accumulation of sorbitol in the polyol pathway, and the excess accumulation of ROS from mitochondrial oxidative phosphorylation. These can also contribute to the inflammatory pathways (NFκB, TNF-α, IL-1, etc.) that may damage cellular components. Created in BioRender. P, V. https://BioRender.com/oyl1i4f (accessed on 21 May 2026).

Peripheral neuropathy is one of the most common long-term complications of diabetes [16]. Patients who develop peripheral neuropathy experience nerve damage leading to pain, weakness, paresthesia, and other nerve disorders typically manifesting in the lower extremities [17]. Chronic hyperglycemic conditions activate several pathways that culminate in morphological changes in nerves and their associated structures (Figure 3).

#### 3.2.1. Polyol Pathway and Oxidative Imbalance

One primary explanatory mechanism is the polyol pathway explained previously [18]. Under normoglycemic conditions, glucose is mainly used in the glycolysis pathway for ATP production. However, in a chronic hyperglycemic state, secondary pathways like the polyol pathway are upregulated to shunt glucose for alternative utilization as glycolytic enzymes become saturated [19]. As alluded to earlier, this leads to the overproduction of ROS. When these ROS remain unchecked in the neural environment, they can cause widespread damage to the structure and function of lipids, proteins, and nucleic acids within the neurons themselves [18].

#### 3.2.2. Mitochondrial ROS and Neural Dysfunction

Specifically, ROS are dangerous with regard to lipid peroxidation. Therefore, they are particularly damaging to the myelin sheaths that surround neurons, which are mainly composed of lipids [18]. This has many implications for the functioning of the nerves. This may lead to the hyperexcitability of nociceptive fibers, spontaneous impulse generation, and decreased conduction velocity in these peripheral nerves [18]. ROS can not only damage neurons and myelin sheaths directly, but they can also lead to nerve dysfunction through an indirect mechanism: excessive ROS generated in chronic hyperglycemic conditions can damage vascular endothelial cells that provide blood flow to these peripheral nerves [6]. These mechanisms were alluded to previously, such as through the disruption of the production of NO and the EndMT process. Thus, damage to those vessels further limits the capacity of these peripheral nerves to function appropriately.

#### 3.2.3. Clinical Management and Therapeutic Approaches

Treatment and management of diabetic neuropathy consist of a multi-step approach. Prevention through education and regular check-ups is crucial. Conservatively, managing the diabetes and hyperglycemia itself through therapies like lifestyle modifications and metformin may also limit the likelihood of development and progression of this complication. For the symptoms of diabetic neuropathy itself, especially in instances of painful neuropathy, pain management measures are taken. This may include duloxetine, pregabalin, amitriptyline, and capsaicin patches to modulate the pain response [20,21]. Future therapies may seek to target the inflammatory response that leads to damage to the nerves and surrounding structures.

## 4. Organ Damage in Diabetes: Microvascular Complications

### 4.1. Diabetic Retinopathy

Diabetic retinopathy (DR) is a serious complication of DM that can lead to vision loss. DR is driven by chronic hyperglycemia, which initiates a cascade of molecular pathways that culminate in vascular permeability, inflammation, cellular damage, edema, and ischemia in the retina.

Hyperglycemic conditions induce diabetic microangiopathy, characterized in part by the thickening of the capillary basement membrane (BM) via the overproduction of extracellular matrix components like collagen IV, fibronectin, and laminin [22]. This altered BM composition has been proposed to disrupt cell–matrix signaling important for pericyte-endothelial adhesion, though the precise contribution of BM changes to pericyte loss remains incompletely defined and is thought to act alongside, rather than independently of, other hyperglycemia-driven insults such as AGE accumulation and hypoxia [23].

#### 4.1.1. AGE–RAGE Signaling, Oxidative Stress, and Early Cellular Injury 

Another key driver of DR is the accumulation of AGEs, which bind to RAGE on retinal endothelial cells and cause a surge in ROS within them. Severe oxidative stress in endothelial cells further impairs pericyte adhesion and survival, and the resulting pericyte dropout destabilizes capillary architecture [24].

The oxidative (and nitrosative) stress also upregulates endothelial ICAM-1 [25,26], which drives leukostasis, in which activated leukocytes adhere to and occlude retinal capillaries, releasing pro-inflammatory cytokines such as IL-1β and TNF-α. These cytokines, along with leukocyte adhesion, downregulate the tight junction proteins occludin and ZO-1 and promote apoptosis of retinal endothelial and pigment epithelial (RPE) cells [25,27]. Together, pericyte loss and leukostasis-driven cell death and tight junction disruption promote the breakdown of the blood–retinal barrier (BRB), producing increased vascular permeability and microaneurysm formation. The resulting paracellular leakage of plasma proteins and lipids into the neurosensory retina manifests clinically as fluid accumulation, macular edema, and hard exudates (ring-like yellow deposits formed by lipid-laden macrophages) [22,28]. Macular edema is the primary driver of vision impairment in diabetic patients [27].

#### 4.1.2. Hypoxia, VEGF Signaling, and Neovascularization

As the disease progresses, widespread endothelial death leads to capillary occlusion and significant retinal ischemia. In an attempt to compensate for this hypoxic environment, the retina upregulates hypoxia-inducible factor 1-alpha (HIF-1α), which promotes the transcription of vascular endothelial growth factor (VEGF). Additionally, hyperglycemia-induced increases in DAG synthesis activate PKC, further promoting VEGF secretion [27]. Although this response is intended to restore blood flow, the resulting neovascularization consists of fragile, leaky vessels prone to hemorrhage. Glial cells surround newly formed blood vessels that have grown into the vitreous, leading to fibrous proliferation on the retinal surface. This fibrous tissue then exerts traction on the retina, pulling the neurosensory layer away from the RPEs and causing retinal detachment [29]. This active neovascularization marks the transition to severe proliferative diabetic retinopathy (PDR), posing a significant risk for permanent vision loss. In advanced PDR, new blood vessels can form on the iris (rubeosis), which obstructs aqueous outflow and increases intraocular pressure, a condition known as neovascular glaucoma [30].

#### 4.1.3. Clinical Progression and Standard Clinical Interventions

Currently, mild or non-proliferative DR (NPDR), with noted microaneurysms, is treated by controlling blood glucose and cholesterol levels. For advanced cases such as PDR and diabetic macular edema, the standard treatments attempt to reduce inflammation and vascular leakage via intraocular corticosteroids, NSAIDs, or intravitreal anti-VEGF injections, like ranibizumab [27]. Laser treatment, or panretinal photocoagulation (PRP), prevents abnormal blood vessel growth and is preferred for its long-term effectiveness against PDR. Though the exact mechanism is not completely elucidated, the widely accepted theory is that the laser destroys peripheral photoreceptors, thereby alleviating oxygen demand in the inner retina, which reduces the secretion of VEGF and consequently reduces neovascularization. Surgical intervention, or vitrectomy, is elected in cases of severe retinal scarring or detachment [27,31]. 

Despite their efficacy, these conventional therapies are constrained by the need for repeated interventions, incomplete patient response, and inability to reverse established retinal damage [27].

#### 4.1.4. Emerging and Future Therapeutic Strategies

Several emerging therapeutic strategies have been explored to target the molecular pathways underlying DR. Among antioxidant approaches, natural compounds such as curcumin, resveratrol, and docosahexaenoic acid (DHA) have demonstrated promising results in animal models, reducing ROS generation, apoptosis, basement membrane thickening, and VEGF release [27,32]. Regarding DHA specifically, postmortem analyses of diabetic human donors revealed significant reductions in retinal DHA levels, and supplementation with high-dose DHA combined with carotenoids has been shown to improve macular function within three months in asymptomatic NPDR patients [27,33].

Aldose reductase inhibitors, like sorbinil or plant-derived from *Ocimum sanctum*, offer another avenue for disrupting polyol pathway activation and increasing endogenous antioxidant defenses [27,34]. However, a clinical trial using sorbinil failed to demonstrate a clinically significant effect on the progression of retinopathy over a 30-month period, leaving the overall efficacy of the class in question [27,35].

The hyperglycemia-induced DAG-PKC-VEGF pathway also presents a therapeutic target. Ruboxistaurin is a PKC-β inhibitor that interrupts DAG-PKC pathway activation and subsequent VEGF production, thereby reducing vascular leakage and pericyte dysfunction [32]. However, despite a New Drug Application submitted by Eli Lilly in 2006 and a following approvable letter from the FDA, ruboxistaurin was never approved due to the requirement for an additional long-term clinical trial, and it remains without FDA approval for any medical use [32,36].

Lipid-modulating agents, such as fenofibrate and PCSK9 inhibitors, represent another promising class of therapies. Fenofibrate significantly reduced the need for laser treatment in DR patients in the multinational FIELD study, and the ACCORD-Eye study reported a reduction in DR progression over four years with fenofibrate use [27]. PCSK9 inhibitors such as evolocumab have demonstrated anti-inflammatory effects in retinal Müller cells through the inhibition of the TLR-4/NF-κB signaling pathway, and serum PCSK9 levels have been positively correlated with advanced DR stages [27].

Antiplatelet therapy has also been explored, with the Dipyridamole, Aspirin, Microangiopathy of Diabetes (DAMAD) study demonstrating that the combined use of aspirin and dipyridamole produced a significant reduction in microaneurysm formation [32,37]. 

Neuroprotective approaches are gaining traction as well. For example, Huang et al. identified peroxiredoxin 4 (PRDX4) as a critical regulator of oxidative and endoplasmic reticulum stress [38]. PRDX4 deficiency exacerbates retinal neurodegeneration, mitochondrial dysfunction, and gliosis under hyperglycemic conditions, whereas its overexpression confers protective effects, reinforcing that enhancing endogenous antioxidant defense systems may be a viable therapeutic avenue [27,38].

Given the multifactorial nature of DR, combination strategies that simultaneously target oxidative stress, inflammation, metabolic dysregulation, and neovascularization are increasingly being recognized as the most promising path forward for comprehensive disease management.

### 4.2. Diabetic Nephropathy

Because they are highly perfused at rest, the kidneys are highly susceptible to chronic hyperglycemic conditions. As a result, diabetes is the leading cause of end-stage renal disease [39]. Due to long-term renal dysfunction, patients with diabetic nephropathy are at higher risk of cardiovascular mortality and morbidity [39]. Clinical diagnosis is dependent on the patient’s estimated glomerular filtration rate (eGFR) and albuminuria. This is a result of structural damage to the filtration barrier within the glomerulus, which includes wrinkling of the basement membrane, glomerular ischemia, glomerulosclerosis, and periglomerular fibrosis [40]. Several mechanisms can be investigated for their possible role in diabetic nephropathy.

#### 4.2.1. Inflammatory and AGE-RAGE Signaling

One of the proposed explanations for the pathogenesis of diabetic nephropathy is through the inflammatory pathways. Chronic hyperglycemia-induced AGE-RAGE interaction leads to downstream cytokine release, such as TNF-α [41]. TNF-α then serves as a mediator of the inflammasome complex NLRP3. This complex activates IL-1β and IL-18 in addition to cell death via pyroptosis [42]. Inappropriate cell death of the glomerular filtration barrier through the inflammatory pathway can compromise its integrity and mediate the degeneration of kidney function seen in diabetic nephropathy.

#### 4.2.2. Oxidative Stress and Senescence

Another pathway that may explain the role of diabetes in kidney disease is cell senescence. In chronic hyperglycemia, increased flux through the oxidative phosphorylation pathway leads to the disproportionate production of ROS [43]. Not only can ROS damage lipid membrane components and proteins, but they can also damage DNA directly [44]. DNA damage to the highly metabolically active glomerular and renal tubular epithelial cells leads to telomere shortening and cell senescence [45]. The presence of these senescent cells, which are resistant to apoptosis, triggers inappropriate tissue fibrosis [46]. This damage to the cells of the glomerulus and the tubular epithelium helps give clarity to the morphological changes seen in diabetic nephropathy.

#### 4.2.3. Glomerular Hyperfiltration

Another proposed mechanism of renal insult from diabetes is glomerular hyperfiltration. This occurs when the additive filtration forces in and around the glomerulus lead to higher rates of filtration. One way is through the renin–angiotensin–aldosterone system (RAAS). The kidneys are regulators of the RAAS, which mediates many vascular effects in order to control blood pressure. In hyperglycemic conditions, though, this system can become upregulated [47]. One of the downstream effects of angiotensin II is to vasoconstrict the efferent arteriole, increasing hydrostatic pressure in the glomerular capillaries. In addition to the upregulation of RAAS in diabetes, proximal tubule reabsorption is increased. Due to the increased load of glucose flowing through the nephron in hyperglycemia, the SGLT is upregulated via increased substrate availability, and a concurrent increase in the activity of the sodium–hydrogen exchanger (NHE3) [48]. Due to the decreased sodium concentration in the tubular fluid, the macula densa causes vasodilation of the afferent arteriole, further increasing the hydrostatic pressure in the glomerular capillaries according to the tubuloglomerular feedback system [49]. This physical increase in the pressure gradient may provide insight as to the structural damage seen in diabetic nephropathy [48].

#### 4.2.4. Therapeutic Approaches

Management of diabetic nephropathy includes primary control of glycemic conditions, such as through lifestyle modifications and metformin. Furthermore, SGLT2 inhibitors are utilized to improve glycemic control and preserve kidney function. In addition, as vascular insults have been suggested in diabetic nephropathy, Angiotensin-Converting Enzyme (ACE) Inhibitors and Angiotensin Receptor Blockers (ARBs) are prescribed to reduce the damage of hyperglycemia to the renal vasculature. As the RAAS was also involved in the pathogenesis of diabetic nephropathy, Mineralocorticoid receptor antagonists (MRAs) may also be prescribed to protect kidney function [50]. Future therapies may seek to target the inflammatory pathways implicated in damage to the glomerular apparatus and mitigate the oxidative stress seen in the highly metabolically active cells found along the nephron. In addition, therapies targeted at better, long-term glycemic control may be useful in the prevention of the onset of renal damage in the first place.

### 4.3. Hearing Loss (Emerging Complication)

Diabetic patients are twice as likely to experience hearing loss as non-DM patients [51].

There is a strong association between DM and sensorineural hearing loss, and various pathological changes have been observed in DM patients’ inner ear [52,53]. These changes include thickening of the spiral modiolar artery, atrophy and decreased total area of the stria vascularis, decreased number of outer hair cells in the organ of Corti, and the atrophy of the spiral ganglion [52,53,54,55].

However, exact mechanisms for these changes are unclear, and whether these changes come about due to diabetes mellitus itself or due to its associated complications, such as coronary artery disease and nephropathy, is not well established [56].

The cochlea is heavily vascularized, and the stria vascularis in the cochlea uses the blood flow to maintain the endocochlear potential that is necessary for sound transduction. It has been proposed that the disruption of blood flow due to diabetic microangiopathy damages the stria vascularis due to reduced oxygen supply and destabilizes the endocochlear potential. Along with reduced oxygen delivery, AGE accumulation contributes to excess ROS production in highly metabolic cells like cochlear hair cells and the spiral ganglion neurons, leading to atrophy and cell death [54,55,57]. 

There have been reports of DM affecting vestibular sense as well, with elderly patients (>65 years old) with DM having a higher incidence of falls compared to healthier elderly patients [58]. Some proposed mechanisms for this change, based on studies performed using animal models of DM, include changes in vasculature and damage to the vestibulocochlear nerve due to myelin glycosylation and ROS in the setting of chronic hyperglycemia [59,60]. Type 1 hair cells, which convert rotational movements into neural impulses in the vestibular system, were shown to be damaged in both type 1 and 2 DM patients, but no microangiopathy was noted in those patients [61].

The exact pathophysiology of diabetic vestibular damage remains unclear, and a definitive causal relationship has yet to be established. Current evidence is largely derived from animal models and small patient cohorts, and dedicated studies directly assessing peripheral vestibular function in DM patients are limited [59,60]. Until prospective analyses are conducted on larger, more homogeneous cohorts, vestibular dysfunction remains a poorly understood complication of diabetes that requires further investigation to distinguish its effects from the confounding roles of neuropathy and retinopathy [59,60,62].

#### Therapeutic Approaches for Diabetic Hearing Loss

The current standard of care for diabetes-related hearing and vestibular impairment emphasizes strict systemic glycemic control and management of diabetic comorbidities (e.g., neuropathy, CAD), proactive clinical screening, and functional rehabilitation to prevent debilitating secondary complications. Recognizing the high prevalence of otodegeneration in diabetic patients, the American Diabetes Association (ADA) formally recommends routine audiological evaluations and the incorporation of ear health into comprehensive care plans [63]. Management fundamentally hinges on intensive glycemic control to minimize ongoing microangiopathic and neuropathic damage to the stria vascularis and vestibulocochlear pathways [63]. For vestibular dysfunction, particularly in diabetic patients with risk of falls, clinical guidelines recommend annual balance screenings and the evaluation of common comorbid conditions like benign paroxysmal positional vertigo [62]. Functional management centers heavily on Vestibular Rehabilitation Therapy, a specialized branch of physical therapy utilizing tailored gaze-stabilization, habituation, and balance exercises to retrain central sensory processing, supplemented by targeted falls-risk counseling and home safety modifications to aggressively mitigate the heightened risk of falls in the aging diabetic population [64].

## 5. Organ Damage in Diabetes: Macrovascular Complications

### 5.1. Cardiovascular Disease

#### 5.1.1. Diabetic Dyslipidemia & Atherosclerosis 

Dyslipidemia is highly prevalent in diabetic patients and is a major risk factor for cardiovascular disease (CVD). Although dyslipidemias are of many types, atherogenic dyslipidemia is the most prevalent in diabetic patients. This involves elevated triglycerides and small dense LDL levels with decreased high-density lipoprotein (HDL) levels [65]. Moreover, it is noted that though low-density lipoprotein (LDL) is a major risk factor for CVD, it is the small dense LDL (sdLDL) that is of major concern in diabetes due to its atherogenicity [66].

The primary mechanism due to which these lipid derangements occur is insulin resistance. Peripheral insulin resistance causes elevated release of free fatty acids (FFAs) from adipose tissue, which are taken up by the liver, thus increasing triglyceride (TAG) synthesis in the liver. Elevated FFAs increase hepatic TAG availability, leading to production of very-low-density lipoprotein cholesterol (VLDL) and by increasing post-translational stabilization of ApoB in the liver. Under normal physiological conditions, insulin promotes ApoB degradation via PI3K activation; however, insulin resistance prevents it. Furthermore, cholesteryl ester transfer protein (CETP) and hepatic lipase play an important role in the formation of sdLDL from VLDL. CETP mediates transfer of triglycerides from VLDL to LDL in exchange for cholesteryl esters, producing triglyceride-rich LDL, which is further converted to sdLDL by hepatic lipase [66]. As mentioned earlier, sdLDL plays a major role in atherogenicity, for which a number of mechanisms have been noted. These include low-affinity LDL receptor, facilitated entry into the arterial wall, greater arterial retention due to increased binding to proteoglycans and greater susceptibility to oxidation [67].

Oxidation of LDL ultimately shifts from its recognition by LDL receptors to recognition by scavenger receptors that cause their entry into macrophages [68]. Accumulation of oxidized LDL in macrophages leads to a foamy cytoplasmic appearance, thus given its name foam cells [69]. These foam cells within the vascular wall characterize the early stage of atherosclerotic lesion, called fatty streak, which also contains vascular smooth muscle cells (VSMCs) and T lymphocytes. These fatty lesions can further progress to atherosclerotic lesions if chronic endothelial injury persists. In later stages, plaques acquire a stable fibrous cap. These atherosclerotic plaques can reduce blood vessel lumen, leading to ischemia and metabolic changes in tissues. If the plaque is unstable, it can induce thrombogenesis, further leading to fatal consequences such as myocardial infarction [70,71].

#### 5.1.2. Therapeutic Approaches in Diabetic Dyslipidemia

Statins are the first-line therapy for diabetic dyslipidemia because they effectively lower LDL cholesterol and reduce cardiovascular risk [72]. However, a significant residual risk remains in many patients due to persistent hypertriglyceridemia and the atherogenic lipid pattern seen in diabetes. In such cases, fibrates may be used as add-on therapy to help lower triglyceride levels and improve overall lipid profiles, particularly in patients with mixed dyslipidemia [73]. Combination therapy has been shown to be more effective than monotherapy in improving lipid abnormalities in diabetic patients with high cardiovascular risk [72].

#### 5.1.3. Endothelial Dysfunction and Thrombosis

T2DM is also one of the major causes of vascular dysfunction and thrombosis in patients, which can cause cardiovascular problems. Diabetic patients have increasingly shown dysfunction in the vascular endothelium. Hyperglycemia promotes vascular dysfunction partly by driving dyslipidemia and oxidative stress, which activates NF-κB, a pro-inflammatory transcription factor involved in endothelial activation and vascular damage [74]. Reduced bioavailability of NO due to oxidative stress and endothelial impairment further limits vasodilation in diabetic vessels, exacerbating vascular dysfunction [75]. This damage ultimately leads to the aggregation of platelets. These pathophysiologic mechanisms serve as key therapeutic targets, as interventions including antiplatelet therapy, statins, and glucose-lowering agents aim to mitigate endothelial dysfunction, reduce oxidative stress, and lower thrombotic risk in patients with diabetes.

Platelets are anucleate cells that play a major role in blood homeostasis and wound healing by forming thrombi or blood clots. Platelets respond to insults to blood vessels, which cause subendothelial collagen exposure and result in their aggregation [74]. However, patients with T2DM experience a higher sensitivity to platelets and overactivity, which has been seen by increased levels of Thromboxane B2 in the urine [76]. Thromboxane B2 is an inactive metabolite of Thromboxane A2, a pro-thrombotic mediator that helps in blood clot formation [74]. Moreover, Plasminogen activator inhibitor-1 is a protein encoded by the SERPINE1 gene, which inhibits the conversion of plasminogen to plasmin. Plasmin is a proteolytic enzyme that helps break down fibrin blood clots, thus being a fibrinolytic enzyme. T2DM patients have been shown to have an increased plasma concentration of PAI-1. However, it has been studied that it is the insulin resistance rather than merely hyperglycemia that leads to increased PAI-1, as patients with T1DM have been shown to have normal plasma concentrations of PAI-1 [77]. Other inflammatory markers such as ET-1, vWF, t-PA, ICAMs, and VCAMs are also released from damaged endothelial cells in T2DM [75]. These changes, along with the association with dyslipidemia, promote atherosclerotic plaque formation and progression. These inflammatory factors also cause structural changes within the atherosclerotic plaque that weaken the protective fibrous cap. This process increases the likelihood of plaque rupture or superficial erosion, triggering thrombus formation upon disruption and contributing to cardiovascular events [70,78,79]. In addition to platelet hypersensitivity, T2DM patients exhibit a hypercoagulable state due to increased fibrinogen levels and reduced anticoagulant factors, further raising the risk of thrombus formation following plaque rupture.

In addition to large vessels being occluded, small coronary vessels are also affected by DM. In fact, studies have shown that coronary microvascular dysfunction is an early finding of T2DM, preceding macrovascular damage [80]. Similar to large vessel endothelial damage, coronary microvascular dysfunction is also related to low levels of NO and increased oxidative stress, leading to impaired vasodilation, causing chest pain, ischemia, and possible infarction [81].

#### 5.1.4. Diabetic Cardiomyopathy and Heart Failure

Heart failure and cardiovascular disorders are considered to be the number one cause of death in diabetic patients. Cardiomyocytes are the muscle cells in the heart that are highly dependent on ATP for excitation–contraction coupling reactions [82]. These coupling reactions are essential for systolic heart function, where an electrical signal leads to Ca^2+^ release and cardiac contraction (pumping blood out). The coupling reactions are also needed for the diastolic phase of the heart, where Ca^2+^ is removed, and the heart relaxes and fills with blood. Thus, ATP is needed for normal heart rhythm and coordinated function. Nearly 95% of ATP in cardiomyocytes is produced by oxidative phosphorylation in mitochondria. The oxidative phosphorylation reaction is primarily driven by NADH and succinate to continue generating ATP. NADH and succinate are products of the TCA cycle (also called the Krebs cycle), and acetyl-CoA is essential to keep the TCA cycle active. Cells in the body produce acetyl-CoA from three main sources: β-oxidation of fatty acids, decarboxylation of pyruvate from glycolysis, and oxidation of ketone bodies [82].

In diabetic patients, impaired insulin signaling leads to both hyperglycemia and hyperlipidemia due to decreased insulin sensitivity and decreased glucose uptake by skeletal muscles and adipose tissue. Adipose tissue then increases the amount of fatty acid secretion [82]. Cardiomyocytes also have a decrease in insulin signaling, leading to less glucose uptake. This causes a metabolic shift by relying less on glucose as an energy source to generate ATP, and more on fatty acid oxidation. Fatty acid oxidation is less efficient in O_2_ use compared to glycolysis and thus generates less ATP. This metabolic shift with an increase in fatty acid use increases O_2_ demand and decreases efficiency in ATP production for the heart. Lipid buildup in the heart also increases oxidative stress. Previous studies have shown that Diabetic cardiomyopathy (DCM) induced by fatty acids is associated with mitochondrial dysfunction, oxidative stress, and inflammation. However, the exact molecular mechanism of fatty acid-induced inflammation and cell death in DCM is still unclear [83]. Diastolic dysfunction is first observed, and as the disease progresses, systolic dysfunction is noted.

Insulin resistance and hyperglycemia lead to vascular endothelium dysfunction of the coronary artery, leading to coronary artery disease (CAD). This is characterized by increased inflammation and oxidative stress, decreased NO biosynthesis, endothelial-mesenchymal transition, senescence, and even cell death [8].

Moreover, hyperglycemia and metabolic shift in energy utilization prevent optimal utilization of glucose by cardiomyocytes, which leads to myocardial fibrosis. Myocardial fibrosis is characterized by an increase in extracellular matrix proteins, deposition of interstitial collagen, disarrangement of cardiomyocytes, and the remodeling of cardiac structure. This disarrangement of cardiomyocytes makes the cells incapable of regeneration, which is considered the most extensive pathological cause of cardiomyocyte death [84].

The accumulation of extracellular matrix and collagen and the remodeled structure of the heart increase ventricular stiffness, reduce compliance and impair diastolic filling [85]. With time, this facilitates the development and progression of diabetic cardiomyopathy. Uncontrolled persistent myocardial remodeling and fibrosis eventually lead to impaired systolic function and heart failure.

The ongoing process of chronic metabolic and structural changes in the diabetic heart can eventually lead to damage to autonomic innervation, leading to cardiac autonomic neuropathy (CAN).

Patients with CAN generally have higher BMI, HbA1C, and longer duration of diabetes [86]. CAN impairs heart rate regulation and autonomic compensation, which further exacerbates left ventricular dysfunction and increases the risk of heart failure and arrhythmias in patients with T2DM. Out of 60 patients, a total of 53 patients (88.3%) with DM had CAN. Of these, 38.3% showed early CAN, 38.3% showed definite CAN, and 7% showed severe CAN [87]. Identifying diabetic individuals with CAN is important because, if detected early on, comprehensive therapies focusing on lifestyle, glucose management, and cardiovascular risk factors can slow down the progression and potentially reverse the course of CAN [87].

Diabetic patients with myocardial fibrosis, diastolic dysfunction, and CAN have impaired cardiac function, which contributes to an imbalance between myocardial oxygen supply and demand. The heart is unable to compensate for the elevated myocardial oxygen demand due to diastolic dysfunction and impaired cardiac function, further exacerbating myocardial stress and dysfunction. Myocardial cells do not meet energy needs because of the decreased amount of oxygen availability. This leads to hypoxia, metabolic stress, and further injury to the coronary vasculature, thus worsening CAD. Electrical instability is a common result as the excitation-coupling reactions are also affected due to the decreased amount of oxygen availability for ATP production. The mismatch between the myocardial oxygen demand and supply predisposes to ischemia and electrical instability. The combination of structural and functional impairments greatly increases the risk of adverse cardiovascular outcomes, including arrhythmias, pump failure, and mortality in diabetic patients. 

Heart failure is a common presenting cardiovascular complication in diabetic patients and is the most expensive complication because of the high cost of hospitalizations [88]. The likelihood of developing CVD is 1.5-fold higher in patients with DM than in patients without DM, and the likelihood of experiencing CV-related events (e.g., myocardial infarction or stroke) is higher in patients with severe or uncontrolled DM [89].

Stroke is another serious complication of DM, with recent studies showing patients with T2DM having up to a 58% increased risk of stroke compared to non-T2DM individuals, compounded by disease processes of DM such as insulin resistance leading to poor stroke outcomes [90]. Stroke is broadly divided into ischemic and hemorrhagic stroke, yet numerous studies consistently show a higher proportion of ischemic stroke than hemorrhagic stroke in DM patients [91,92]. Therefore, this paper will mainly discuss the cellular mechanisms of diabetes-induced ischemic strokes.

#### 5.1.5. Large Vessel Ischemic Stroke (LVO) Mechanisms in Diabetes

Ischemic strokes in the context of DM can either be due to large vessel occlusion (LVO), such as carotid or intracranial arteries, or microvasculature in the deep brain structures, such as the lenticulostriate artery leading to lacunar stroke, a subtype of ischemic stroke. While LVOs are more prevalent in the general population, patients with DM are also susceptible to lacunar strokes due to the disease processes of DM [93]. Large and small vessel strokes share similar upstream cellular mechanisms but lead to different downstream effects, leading to distinct pathological processes [94]. The overlapping molecular players between LVOs and lacunar strokes include PKC, polyol, and AGE-RAGE pathways stimulated by chronic hyperglycemia in DM, all of which collectively increase inflammation and ROS stress, ultimately leading to endothelial dysfunction as discussed in the earlier section. In macrovascular strokes, the endothelial dysfunction accelerates a buildup of atherosclerotic plaques, which could eventually rupture and occlude one of the large vessels supplying the brain, such as the vertebrobasilar artery, leading to LVO [94]. Hyperglycemia upregulates metalloproteinase production by macrophages, which in turn destabilize atherosclerotic plaques and expose the lipid core to activate prothrombotic processes. This ultimately leads to plaque rupture and increases the likelihood of a thromboembolic event [92].

#### 5.1.6. Small Vessel Disease and Lacunar Stroke in Diabetes

The same diabetes-induced cellular mechanisms, such as polyol and PKC pathways, which culminate in endothelial dysfunction, have distinct downstream outcomes in the penetrating arterioles perfusing deep brain structures, leading to lacunar stroke. Specifically, pericyte loss, basement membrane thickening, and lipohyalinosis are regarded as crucial in the pathogenesis of diabetes-induced lacunar strokes [91,92,94]. The oxidative stress and inflammation at the small vessel level can lead to ECM remodeling and damage, causing pericyte loss and thickening of the basement membrane. As DM progresses, the constant stress to small vessels could potentially cause leakage of plasma protein into the arteriolar wall media, a process known as lipohyalinosis, leading to hyaline deposition and therefore occlusion of a small vessel. Lipohyalinosis has proven to be a major cause of lacunar stroke, but the exact mechanism by which diabetes causes lipohyalinosis has not yet been delineated [92].

#### 5.1.7. Therapeutic Approaches for Stroke Prevention in Diabetes

Several pharmacological therapeutic options for diabetes have been shown to significantly reduce the risk of diabetes-induced stroke. Particularly, GLP-1 receptor agonists are actively being explored in the context of reducing cardiovascular complications in patients with diabetes. A recent systematic review by Garcia-Casares et al. demonstrated the efficacy of Semaglutide and dulaglutide in reducing stroke incidence in DM patients [95]. Similarly, a recent meta-analysis also showed a significant decrease in the likelihood of ischemic strokes in DM patients [96]. Another therapy currently investigated for the protective effect of diabetes-induced stroke is SGLT-2 inhibitors. In contrast to GLP-1 receptor agonists, SGLT-2 inhibitors only demonstrated a significant reduction in cardiovascular events but did not affect stroke in DM patients [97].

## 6. Impaired Healing and Immune Dysfunction

### 6.1. Chronic Wounds and Diabetic Foot Ulcers

Chronic wounds represent a significant complication of diabetes, arising from impaired healing processes driven by hyperglycemia-induced vascular, inflammatory, and cellular dysfunction.

#### 6.1.1. Susceptibility to Wounds

Patients with DM are more susceptible to the formation of chronic wounds [98]. Diabetic hyperglycemia is a factor affecting inflammation, angiogenesis, atherosclerosis, neuropathy, and wound healing [98,99,100]. Wounds heal in a series of complementary and overlapping stages that include hemostasis, inflammation, proliferation, and remodeling [99,100].

In the first stage, degranulation of mast cells after an injury causes increased capillary permeability and vasodilation, allowing more transportation of immune cells to the site of injury. Coagulation occurs as platelets lay a network of insoluble fibrin. Inflammatory mediators are also released and promote the response of the immune system. In patients with DM, the hyperglycemic environment can contribute to atherosclerosis and impair the transport of nutrients to the sites of injury [98].

**Figure 4:**
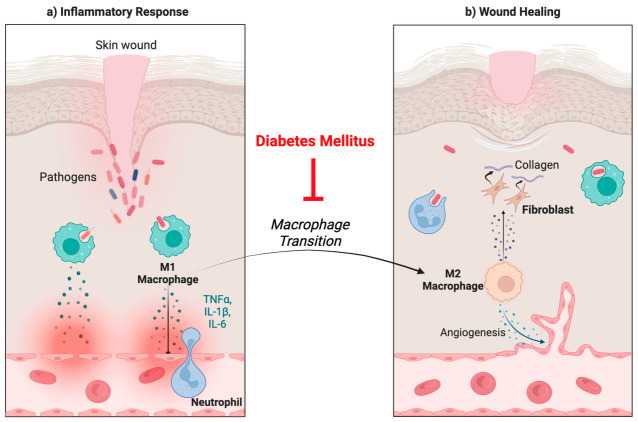
Dysregulation of Macrophage Differentiation in Diabetic Wound Healing. (**a**) In normal healing, M1 macrophages phagocytose pathogens and debris near the wound site, and they also secrete inflammatory cytokines. (**b**) Differentiation of the macrophage from the M1 to the M2 phenotype usually occurs around day 7. These macrophages secrete anti-inflammatory cytokines and promote angiogenesis and collagen deposition, leading to wound healing. In diabetics, this phenotype switch is attenuated, leading to prolonged M1 macrophage action resulting in chronic inflammation and slower wound healing. Created in BioRender. P, V. https://BioRender.com/d5l9ger (accessed on 21 May 2026).

The influx of inflammatory mediators starts the inflammatory phase, where immune cells like macrophages, neutrophils, and monocytes respond to the site of injury to clean debris, damaged cells, and microbes. There is an accumulation of cytokines and chemokines, which further attract monocytes to respond. Although this inflammatory response is a normal part of the healing process, it is prolonged in diabetic patients, which impairs the healing process. In healthy patients, monocytes differentiate into macrophages, and when exposed to danger-associated molecular patterns (DAMPs) from tissue damage, they differentiate into M1 macrophages, which secrete cytokines like interleukin 6 (IL-6) and TNF-α that regulate early healing (Figure 4). 

In early repair, the pro-inflammatory M1 makes up the majority, with the anti-inflammatory macrophages being the majority later in the process [99]. In diabetic patients, there is a dysregulation of the inflammatory phase contributing to wounds like ulcers. There is a reduced ability to phagocytose pathogens and a reduced ability for the macrophages to differentiate into the M2 phenotype, leading to the next phase of repair [99]. The reduction in M2 macrophages causes a deficiency in anti-inflammatory cytokines, which prolongs the inflammation phase observed in diabetic wounds [100]. This process has been supported in mouse models that show that the switch from M1 to M2 macrophages usually occurs 7 days after initial injury, but it is not seen in diabetic environments [101].

After the inflammatory phase is the proliferative phase, involving re-epithelialization. Macrophages from the inflammatory phase are involved in the stimulation of keratinocytes, fibroblasts, and endothelial cells to proliferate [99]. Granulation tissue, consisting of fibroblasts, immune cells, and newly formed blood capillaries, is critical for the process of re-epithelialization. In a patient with diabetes, the prolonged inflammatory phase and deficiency of M2 macrophages impair the healing process and lead to chronic wounds [100]. Attenuated angiogenesis is another factor involved. It is known that the formation of new blood vessels is critical for the healing process of wounds.

Revascularization via angiogenesis is critical for the healing of wounds, and it relies on endothelial progenitor cells (EPCs) from bone marrow [102]. Fadini et al. demonstrate that diabetic patients have a 40% mean reduction in peripheral EPC and that peripheral vascular disease was associated with low EPC levels [103].

Wound remodeling is the fourth stage of the healing process. The granulation tissue forms scar tissue, and M2 macrophages continue to play a critical role in remodeling. Collagen III is degraded and replaced by type I collagen mediated by TGF-β and FGF signaling [100]. Fibroblasts and macrophages shape the final structure of the healing wound as the collagen fibers become denser. In patients with DM, the reduced angiogenesis results in a hypoxic environment causing oxidative stress [98,100].

#### 6.1.2. Foot Ulcers

One of the most common wounds that diabetic patients develop is diabetic foot ulcers (DFUs). A diabetic foot ulcer is defined by the International Working Group on the Diabetic Foot (IWGDF) as a break in the skin of the foot that involves, as a minimum, the epidermis and part of the dermis and affects a person currently or previously diagnosed with diabetes mellitus [104]. Approximately 15% of people with diabetes develop foot ulcers, and around 14–24% require amputation [100,105]. It is estimated that every 30 seconds, a person somewhere in the world loses a limb due to diabetes [106]. This prevalence causes a financial burden, as it was estimated that DFU and amputations cost US health payers $10.9 billion in 2001 [106].

In addition to what has been previously mentioned on wounds, the pathophysiology of DFUs involves the interplay between neuropathy, vascular insufficiency, and infection [105]. Peripheral neuropathy is seen in approximately 10.9–32.7% of diabetic patients in the US [107]. Neuropathy leads to a loss of sensation in the feet, which causes minor trauma to go unnoticed in diabetic patients [105,107,108]. This trauma, if unattended, leads to ulceration, which is a major risk for diabetic foot infection (DFI) from opportunistic pathogens [107,108]. DFIs increase the risk of limb amputation by 50% for patients with DFUs [108]. The loss of sensation occurs due to glucose products that accumulate, namely fructose and sorbitol, causing osmotic stress that impairs nerve conduction by diminishing myoinositol synthesis [105]. Another complication the diabetic patient faces is autonomic neuropathy impairing the neuronal control of sweat glands, leaving dry skin susceptible to fissures, pruritus, and infections [98]. Vascular insufficiency stems from impaired angiogenesis as mentioned previously. Infection further progresses the ulcer and impairs its healing, often leading to amputation to prevent its spread [107].

#### 6.1.3. Treatments

Many treatment strategies have been explored with efforts in managing chronic wounds, including DFUs. Standard treatments include debridement, dressings, antibiotics, and others [109]. Advanced treatments still being researched to see if they perform better than the standard include, but are not limited to, stem cell therapy, ozone therapy, dermal matrices, and skin substitutes [109,110,111]. The current literature in this area consists of systematic reviews analyzing early clinical data as well as preclinical mechanisms [109,110,111]. 

Among the stem cell therapies being researched, adipose tissue mesenchymal stem cells (AT-MSCs) are often used for tissue regeneration and are seen as optimal due to their proliferative abilities as described in literature reviews [110,111]. They have also shown expression of angiogenic factors such as VEGF, insulin-like growth factor-1 (IGF-1), and interleukin 8 (IL-8), making it a possible therapeutic option in patients with DM [110]. Another stem cell alternative is bone marrow-derived mesenchymal stem cells (BM-MSCs), which recent systematic review data show have had positive effects, such as wound size reduction in patients [111]. Stem cell therapy is a potential treatment for managing chronic wounds in diabetic patients, and further large-scale clinical trials are required to establish standard protocols for treatment.

Another potential treatment being considered for DFUs is ozone therapy. It involves the use of ozone gas, which is an oxidant, but more mechanistic and clinical research needs to be performed to understand its mechanisms in contributing to the healing of wounds [109]. Currently, a recent systematic review suggests that ozone therapy can have positive effects, although there is still disagreement in this area, and more needs to be known about its safety [109].

Skin substitutes are another modality of treatment being explored for their ability to promote revascularization and assist healing of wounds like DFUs. Among these skin substitutes is the use of acellular dermal matrices (ADMs). Clinical studies have shown that ADMs can reduce the time to heal a wound when compared to the standard treatments, and it does not generate more complications [112]. Another similar treatment that has been shown to provide positive benefits is the use of dermal replacement therapy, which helps promote angiogenesis and accelerate the healing process of DFUs [113].

### 6.2. Immune System Dysfunction

Diabetes is associated with significant immune system dysfunction, characterized by chronic inflammation, impaired innate immunity, and increased susceptibility to infection. 

#### 6.2.1. Inflammation

Individuals with diabetes mellitus are in a state of hyperglycemia, which, as discussed previously, subjects them to a state of chronic low-grade inflammation. This state subjects the person to a combination of metabolic stress, oxidative stress, and cytokine abnormalities. It has been observed that, in people with diabetes, there are increased levels of cytokines like IL-6, TNF-α, and IL-1β, which are inflammatory markers contributing to the chronic inflammation and insulin resistance [114,115]. Investigations have been performed to target these cytokines for anti-inflammatory treatment, which will be discussed later. 

People with diabetes are more susceptible to wounds, leading to more inflammation during the healing process. Wounds make diabetics more prone to infections, and due to changes in cytokines and macrophages, this risk is further increased [116]. Research indicates that a state of hyperglycemia impairs macrophage phagocytosis, thus weakening their activity in immune responses [116].

With inflammation, the production of ROS follows, and it has been shown to be increased in individuals with diabetes mellitus [117]. As described above, in hyperglycemic conditions, excess glucose forms AGEs and is also shunted through the polyol pathway, producing osmotic stress and depleting the cellular pool of NADPH [117,118]. Depletion of NADPH prevents the formation of a functional GSH to act as an antioxidant against ROS and contributes to the accumulation of ROS seen in diabetic individuals [118].

In addition to the increase in ROS, inflammation from hyperglycemia induces the senescence of bone marrow stem cells, which express senescence-associated secretory phenotypes (SASPs) that include IL-1ɑ, IL-1β, IL-6, and NF-κB [117]. These markers are themselves pro-inflammatory, contributing to the state of chronic inflammation seen in diabetic patients [117].

#### 6.2.2. Treatments

With chronic inflammation from hyperglycemia being involved in many of the issues seen in diabetic individuals, much research has been conducted on how to combat this inflammation. Most potential treatments are looking to target pro-inflammatory cytokines while decreasing ROS. One such treatment is the use of GLP-1 receptor agonists, which are already in use as a treatment for T2DM [119]. Recent research shifts focus to its role in anti-inflammatory action and how those could be an additional mechanism in the treatment of diabetes [119]. Additionally, research involving mice indicates that GLP-1 receptor agonists protect beta cells in pancreatic islets from inflammatory stress [120]. From what has been discussed before, reducing the chronic inflammation seen in diabetes would be beneficial in the management of the disease, making these treatments an area of focus.

A major marker of inflammation, as previously mentioned, is IL-6, making it a target of some treatments in order to reduce its effects. One such treatment involves physical exercise with the goal of increasing “exerkines”, molecules released due to exercise-related changes, to positively affect health [121,122]. As García-Hermoso et al. demonstrated in their systematic review and meta-analysis, exercise training can reduce pro-inflammatory markers including IL-6, which may reduce complications in people with diabetes [122].

Other methods of reducing oxidative stress and thus improving immune system function in diabetic patients include the use of curcumin and senolytics. Curcumin is an anti-inflammatory agent that can reduce oxidative stress and can be used in conjunction with other treatments to improve outcomes in diabetics [123]. Research is still being performed on how to optimize its delivery for effective treatment due to its insolubility [123]. Senolytics are compounds that target senescent cells, which, as previously mentioned, contribute to the state of inflammation affecting the immune function in diabetic patients [124]. Although there is some promise for senolytics in being able to reduce diabetic complications, more studies are needed to establish if it is an effective therapeutic [124].

## 7. Conclusions

Diabetes mellitus is a common metabolic disorder that causes extensive cellular and molecular dysfunction in many organ systems. As shown in Figure 2, hyperglycemia simultaneously activates oxidative stress, AGE-RAGE signaling, the polyol pathway, PKC signaling, chronic inflammation, and endothelial-to-mesenchymal transition, with each pathway amplifying the others through a positive feedback mechanism. Despite their diverse molecular origins, these pathways ultimately converge on endothelial dysfunction, neuropathy, impaired tissue perfusion, and inflammation. Because every organ depends on an intact vascular and neural network, these shared mechanisms provide a common pathological foundation for diabetic complications throughout the body. The specific clinical manifestation that develops, whether neuropathy, retinopathy, nephropathy, CVD, stroke, impaired wound healing, or hearing loss, is largely determined by the structural and metabolic characteristics of the affected tissue. 

Due to these common inflammatory pathways precipitating from chronically elevated blood-glucose levels, many of the first-line treatments, such as lifestyle or diet modifications, metformin, SGLT-2 inhibitors, and GLP-1 receptor agonists, focus on glycemic control. However, the primary action of these therapies is to target upstream metabolic dysregulation, but they do not fully reverse chronic tissue damage. This emphasizes the importance of developing novel drugs that more specifically target molecular pathways, including oxidative stress and inhibition of AGE-RAGE signaling. Emerging therapeutics seek to target these pathways, rather than the hyperglycemia itself. For example, DHA supplementation, curcumin, and resveratrol may target the production and damaging effects of ROS. Additionally, aldose reductase inhibitors might be useful in the disruption of sorbitol buildup in the polyol pathway. Other therapeutics, like PCSK9 inhibitors, may be useful in the pathogenesis of diabetic endothelial damage. Ruboxistaurin may also be potentially useful in diabetic angiogenesis and the resultant vascular leakage. In diabetic foot ulcers, new matrices and stem cell therapy may assist in the wound healing process (Table 1).

Future research should focus on finding strategies that help detect and prevent irreversible tissue damage. Simultaneously targeting a combination of multiple pathogenic pathways is of greater benefit to help reduce diabetic complications and provide more effective treatment. This will aid in reducing the global burden of diabetes mellitus and in improving patient outcomes.

## Figures and Tables

**Table 1:** Summary of major mechanisms of diabetes-induced organ damage and therapies.

Organ System	Mechanism of Diabetes- Induced Damage	Major Molecular Pathways	Current Therapies	Emerging Therapies
Vasculature and Endothelium	Inflammation Oxidative Stress and Reactive Oxygen Species (ROS) Endothelial-to-Mesenchymal Transition (End-MT)	AGE-RAGE activating NF-κB pathway leading to inflammation Hyperglycemia-induced production of electron-donating products generating excess ROS Chronic hyperglycemia stimulates Protein Kinase C (PKC), phosphorylating NADPH oxidase to produce more ROS Canonical Smad 2/3 pathway from hyperglycemia induces gene products leading to End-MT	GLP-1 Receptor Agonists SGLT-2 inhibitors Metformin	
Central Nervous System (Stroke)	Large Vessel Occlusion (LVO): Blockage of major arteries due to atherosclerotic plaques Small Vessel Disease and Lacunar Stroke: pericyte loss, basement membrane thickening, and lipohyalinosis	Endothelial dysfunction from hyperglycemic-induced processes such as PKC, polyol, and AGE-RAGE pathway causes atherosclerotic plaque buildup	GLP-1 receptor agonists	SGLT-2 inhibitors
Diabetic Retinopathy	NPDR and Diabetic Macular Edema (DME): - Inflammation, BM thickening, pericyte dropout, leukostasis, and tight junction degradation leading to BRB breakdown and vascular leakage. **PDR and Advanced** Complications (Detachment, Rubeosis): Capillary occlusion causing severe ischemia, pathological neovascularization, fibrovascular tissue traction, and aqueous outflow obstruction.	AGE/RAGE, ROS, PKC, IL-1β, TNF-α, HIF-1α, VEGF	NPDR and DME: Systemic metabolic control, intravitreal anti-VEGF, intraocular corticosteroids, NSAIDs. PDR and Advanced Complications: PRP laser (reduces peripheral oxygen demand), Pars plana vitrectomy (for scarring/detachment).	NPDR and DME: Ruboxistaurin (PKC-β inhibitor), DHA supplementation, aldose reductase inhibitors, natural antioxidants (Curcumin/Resveratrol). PDR and Advanced Complications: Fenofibrate (PPAR-α agonist), PCSK9 inhibitors (evolocumab via TLR-4/NF-κB), PRDX4 overexpression (neuroprotection).
Hearing Loss	Sensorineural Hearing Loss (*Cochlear Damage*): - Microangiopathy of the spiral modiolar artery causing stria vascularis hypoxia - Disruption of the endocochlear potential - Outer hair cell and spiral ganglion atrophy Vestibular Dysfunction (*Balance Loss and Falls*): - Demyelination and damage of the vestibulocochlear nerve - selective degradation of Type 1 vestibular hair cells	AGEs-RAGE, ROS, Myelin glycosylation	-Systemic glycemic control, management of comorbidities (CAD, nephropathy), hearing aids/amplification. -physical/vestibular rehabilitation, fall-prevention tracking	
Nephropathy	Glomerular filtration barrier basement membrane fibrosis Glomerular hyperfiltration Overactivation of RAAS	AGEs-RAGE, ROS generation, Polyol pathway	ACE inhibitors, ARBs, MRAs	
Neuropathy	Demyelination	AGEs-RAGE, Polyol pathway, ROS generation, NF-κB inflammatory pathway	Duloxetine, pregabalin, capsaicin patches, pain management measures	
Cardiovascular	Diabetic Dyslipidemia and Atherosclerosis: Insulin resistance increases FFAs, VLDL, and small dense LDL, promoting foam cell formation and atherosclerotic plaque development. Endothelial Dysfunction and Thrombosis: Endothelial injury reduces NO, increases platelet activation and inflammation, promoting thrombosis and coronary microvascular dysfunction. Diabetic Cardiomyopathy and Heart Failure: Increased FA metabolism causes oxidative stress, mitochondrial dysfunction, myocardial fibrosis, ventricular stiffness, and heart failure. Insulin resistance and metabolic shift Endothelial dysfunction and coronary artery disease Myocardial fibrosis and cardiac remodeling Cardiac autonomic neuropathy (CAN) Progression to diabetic cardiomyopathy and heart failure	Insulin resistance and diabetic dyslipidemia Oxidative Stress (ROS) NF-Kb-mediated inflammation ↓ PI3K/Akt/eNOS → ↓ NO Oxidized LDL and foam cell formation ↑ PAI-1 and platelet activation Myocardial fibrosis and mitochondrial dysfunction Impaired insulin signaling Increased fatty acid oxidation Mitochondrial dysfunction Oxidative stress and inflammation Decreased nitric oxide bioavailability Extracellular matrix deposition and fibrosis	Lifestyle modification and glycemic control Statins ± fibrates Antiplatelet therapy Metformin SGLT-2 inhibitors GLP-1 receptor agonists	Combination lipid-lowering therapy Expanded use of SGLT-2 inhibitors for heart failure, diabetic cardiomyopathy GLP-1 receptor agonists for cardiovascular protection [125] Therapies targeting oxidative stress, inflammation, and endothelial dysfunction

## Data Availability

No new data were created in the production of this literature review. The data presented in this study were derived from the following resources available in the public domain(s) of: https://pubmed.ncbi.nlm.nih.gov/.

## Abbreviations

The following abbreviations are used in this manuscript:

ACCORD	Action to Control Cardiovascular Risk in Diabetes
ACE	Angiotensin-Converting Enzyme
ADM	Acellular Dermal Matrices
AGE	Advanced Glycation End products
Akt	Protein kinase B
AS	Atherosclerosis
AT-MSCs	Adipose Tissue Mesenchymal Stem Cells
ATP	Adenosine triphosphate
BH4	Tetrahydrobiopterin
BM	Basement membrane
BM-MSCs	Bone Marrow-derived Mesenchymal Stem Cells
BMI	Body Mass Index
BRB	Blood–Retinal Barrier
CAD	Coronary Artery Disease
CAN	Cardiac Autonomic Neuropathy
CETP	Cholesteryl Ester Transfer Protein
CVD	Cardiovascular disease
DAG	Diacylglycerol
DAMAD	Dipyridamole, Aspirin, Microangiopathy of Diabetes
DAMPs	Danger-Associated Molecular Patterns
DCM	Diabetic cardiomyopathy
DFI	Diabetic Foot Infection
DFUs	Diabetic Foot Ulcers
DHA	Docosahexaenoic acid
DM	Diabetes Mellitus
DR	Diabetic Retinopathy
ECM	Extracellular matrix
eGFR	Estimated Glomerular Filtration Rate
EndMT	Endothelial to Mesenchymal Transition
eNOS	Endothelial Nitric Oxide Synthase
EPCs	Endothelial Progenitor Cells
ET-1	Endothelin-1
ETC	Electron Transport Chain
FADH2	Reduced Flavin Adenine Dinucleotide
Ffa	Free fatty acids
FGF	Fibroblast Growth Factor
FIELD	Fenofibrate Intervention and Event Lowering in Diabetes
FMR	Functional Mitral Regurgitation
GLP-1	glucagon-like peptide-1
GLUT4	Glucose Transporter Type 4
GSH	Glutathione
Hb	Hemoglobin
HDL	High-density lipoprotein
HIF-1α	Hypoxia-Inducible Factor 1-alpha
ICAM-1	Intercellular Adhesion Molecule 1
IGF-1	Insulin-like Growth Factor-1
IL-18	Interleukin 18
IL-1β	Interleukin-1 beta
IL-6	Interleukin 6
IL-8	Interleukin 8
IWGDF	International Working Group on the Diabetic Foot
LDL	Low-Density Lipoprotein
LV	Left Ventricle
LVO	Large Vessel Occlusion
MAPK	Mitogen-Activated Protein Kinase
MRAs	Mineralocorticoid receptor antagonists
NADH	Nicotinamide Adenine Dinucleotide (reduced)
NF-kB	Nuclear factor kappa-light-chain-enhancer of activated B cell
NHE3	Sodium–Hydrogen exchanger
NLRP3	NLR family pyrin domain containing 3
NO	Nitric Oxide
NOX	NADPH oxidase
NPDR	Non-Proliferative Diabetic Retinopathy
PAD	Peripheral arterial disease
PAI-1	Plasminogen Activator Inhibitor-1
PCSK9	Proprotein Convertase Subtilisin/Kexin Type 9
PDR	Proliferative diabetic retinopathy
PI3	Phosphoinositide 3-kinase
PKC	Protein Kinase C
PRDX4	Peroxiredoxin 4
PRP	Panretinal photocoagulation
RAAS	Renin–angiotensin–aldosterone system
RAGE	Receptor for Advanced Glycation Endproducts
ROS	Reactive Oxygen Species
RPEs	Retinal pigment epithelial cells
SASPs	Senescence-Associated Secretory Phenotypes
sdLDL	Small dense low-density lipoprotein
SERPINE1	Serpin Family E Member 1
SGLT2	Sodium-Glucose Cotransporter 2
t-PA	Tissue Plasminogen Activator
T2DM	Type 2 Diabetes Mellitus
TAG	Triacylglyceride
TCA	Tricarboxylic acid cycle
TGF-β	Transforming growth factor-beta
TLR4	Toll-like receptor 4
TNF-α	Tumor necrosis factor-alpha
VCAM1	Vascular Cell Adhesion Molecule 1
VEGF	Vascular Endothelial Growth Factor
VLDL	Very-Low-Density Lipoprotein
VSMCs	Vascular Smooth Muscle Cells
vWF	von Willebrand Factor
